# The Application of Differing Machine Learning Algorithms and Their Related Performance in Detecting Skin Cancers and Melanomas

**DOI:** 10.1155/2022/2839162

**Published:** 2022-05-04

**Authors:** Suboh Alkhushayni, Du'a Al-zaleq, Luwis Andradi, Patrick Flynn

**Affiliations:** Minnesota State University, Mankato, USA

## Abstract

Skin cancer, and its less common form melanoma, is a disease affecting a wide variety of people. Since it is usually detected initially by visual inspection, it makes for a good candidate for the application of machine learning. With early detection being key to good outcomes, any method that can enhance the diagnostic accuracy of dermatologists and oncologists is of significant interest. When comparing different existing implementations of machine learning against public datasets and several we seek to create, we attempted to create a more accurate model that can be readily adapted to use in clinical settings. We tested combinations of models, including convolutional neural networks (CNNs), and various layers of data manipulation, such as the application of Gaussian functions and trimming of images to improve accuracy. We also created more traditional data models, including support vector classification, K-nearest neighbor, Naïve Bayes, random forest, and gradient boosting algorithms, and compared them to the CNN-based models we had created. Results had indicated that CNN-based algorithms significantly outperformed other data models we had created. *Partial results of this work were presented at the CSET Presentations for Research Month at the Minnesota State University, Mankato.*

## 1. Introduction

There are three main types of skin cancer: basal cell carcinoma (BCC), squamous cell carcinoma (SCC), and melanoma. Even though melanoma is typically considered the least common form of skin cancer, it causes most cases of skin cancer. According to the statistics from the last few years, melanoma is recognized as the fastest-growing form of skin cancer. The American Cancer Society published that there are about 100,350 American adults (60,190 men and 40,160 women) estimated to have melanoma of the skin. There will be 6,850 adults, 4,610 men and 2240 women, estimated to die from melanoma this year. Current treatment methods for skin cancer include radiation therapy, chemotherapy, and immunotherapies, which can have significant side effects while effective [1].

However, for an effective treatment, early diagnosis of the patient is quite important. Melanoma can grow very quickly if it has not been treated from the early stages. Melanoma can be easily spread to the lower part of the skin, enter the bloodstream, and spread to the other parts of the body. Dermatologists screen the suspicious skin lesion using their expertise for a primary skin cancer diagnosis. They also consider other factors such as the patient's age, lesion's location, nature, and if the lesion bleeds. It is pretty challenging to identify cancerous skin lesions even with this information. Thus, accurate detection is quite critical in providing necessary treatments for the patients and is shown within this work the important role that data models play in diagnosing disease.

Therefore, any acceleration in diagnosing melanoma (and other skin cancers) would likely provide for better outcomes in patient populations. The training and use of a machine learning model, which could provide additional feedback to care providers, would help to simultaneously provide more capacity for screening of patients and allow a care provider to rapidly identify cases that require intervention. The model to be created would likely be a convolutional neural network due to its strengths in the classification of images and the ability to potentially extend the model to include other skin conditions of concern (lesions, gangrene, etc.).

**Figure 1:**
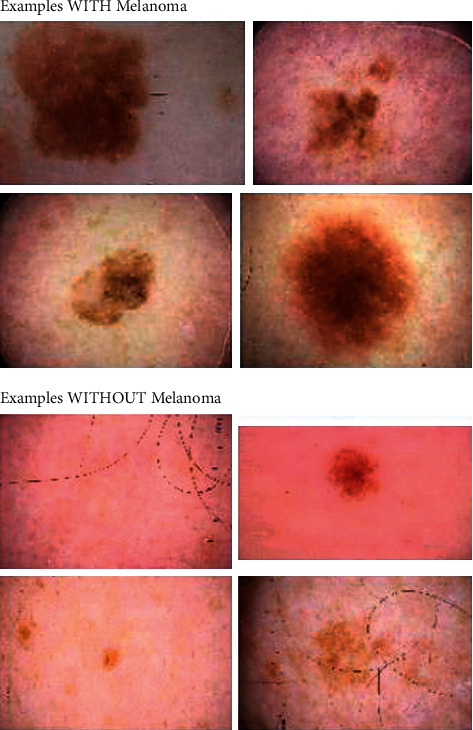
Example with and without melanoma.

Additionally, many researchers struggle to find comprehensive and valid datasets to test and evaluate their proposed techniques, and having a suitable dataset is a significant challenge. Therefore, most studies seem to have fewer than 5000 datasets with neural network [2]. The dataset we will use is a freely available Society for Imaging Informatics in Medicine (SIIM-ISIC) melanoma classification dataset. The dataset was generated by the International Skin Imaging Collaboration (ISIC), and images are from the following sources: Hospital Clinic de Barcelona, Medical University of Vienna, Memorial Sloan Kettering Cancer Center, Melanoma Institute Australia, University of Queensland, and the University of Athens Medical School. This dataset contains malignant and benign 33,126 unique images from 2,000 over patients.  Figure 1 shows a sample of the benign and malignant images in the dataset.

Also, most studies did not evaluate their model against any other model. Some researchers' feature extracted from CNN was fed into the traditional classifiers, such as support vector machine (SVM), random forest (RF), K-nearest neighbor (KNN), and Naïve Bayes (NB), to diagnose the skin image.

We built different CNN implementations in this work and compared the performance between these new models and other more traditional models. Our primary metric is accuracy. So, the next section talks about image rescaling and augmentation, which would improve the model accuracy and efficiency. The following section compares the efficacy of various machine learning models as to their ability to detect cancer given a fixed data set. It also talks about the architecture of these models. Finally, the last section discusses the result of this work with the various models.

## 2. Related Works

Skin cancer is one of the most prevalent cancers among humans, and early detection of skin cancer is very important for prevention and treatment. Currently, a very few real-time skin cancer detection systems are available, and the need for such a system is essential.  Table 1 summarizes some related work for different methods (see Table 2).

## 3. Experimental Section

### 3.1. Methodology

**Figure 2:**
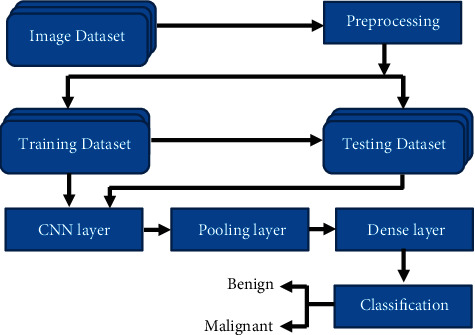
Basic CNN proposed model.

Image rescaling was done on the dataset [10] to normalize the pixel data, and it will improve the model accuracy and efficiency in preprocessing step. Image augmentation, such as changing the image size, image normalization, image rotation, image width shift range, image height shift range, shear range, Gaussian noise, and converting blue, green, and red (BGR) image to lab, and BGR to some other formats, was carried out to have a better identification of malignant and benign masses. Two different folders were created for training and testing and inside each folder created another two different folders for benign and malignant images from the initial data set. There were 584 malignant images and 32,542 benign images in the initial data set. 80% of malignant and benign datasets were used for training, and 20% were used for testing. These two sets were randomly selected and placed in training and testing folders without replacement. Then, 3 different CNN models and one prebuild CNN architecture (VGGNet-16) are created to check the accuracy in image classification. The basic CNN model contains three main layers such as convolutional layer, max pooling layer, and dense layer. Basic CNN proposed model is shown in Figure 2. The convolutional layer applies the output function as a feature map from the image, and the pooling layer was used to reduce the size of the representation and to reduce the speed, which enhances the ability to recognize an object. The fully connected layer transforms the data dimension connecting previous layers to the next layer. The second and third models contain an extra layer: the dropout layer. The dropout layer randomly sets input units to 0 with a rate frequency at each step during training time, which helps prevent overfitting. Accuracy percentage was improved using the image augmentation, changing the hyperparameters, and adding some layers to the CNN models.

The next step is to compare the efficacy of various machine learning models as to their ability to detect cancer given a fixed data set.

### 3.2. Model Definitions

#### 3.2.1. First Model

Model one was created using 3 convolutional neural network layers of increasing kernel size, on a 3px by 3px section of each image. Rectified linear unit (ReLu) is used as an activating function in CNN layers. We then applied a pooling layer to each CNN layer, flattened the layer, and then applied 2 dense layers, using different activation functions (rectified linear unit and sigmoid functions, in that order), giving us a model that is ready to compile. RMSprop uses as the optimizer with 0.0001 learning rate. CNN model one architecture is shown in Table 3.

#### 3.2.2. Two Model

**Figure 3:**
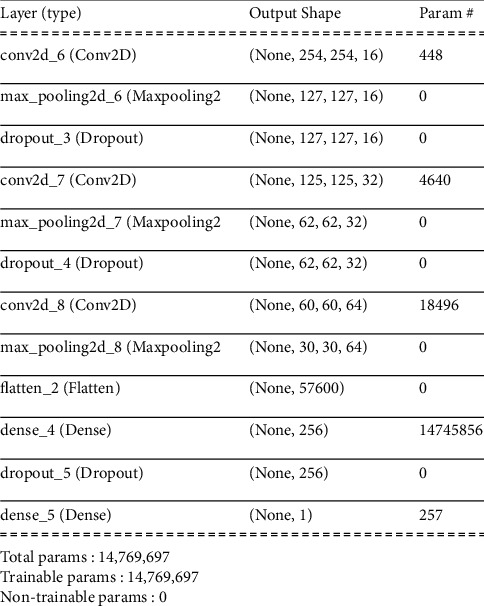
CNN model two architecture from summary () function.

The second model tested made use of image augmentation–rescale (normalize), image size, image rotation, image width shift range, image height shift range, and shear range–to create a more normalized image. Model two has the same layers as layer 1 with the same parameters. Additionally, we added dropout layers after each pooling layer and the first dense layer. CNN model two architecture is shown in Figure 3.

#### 3.2.3. Third Model

**Figure 4:**
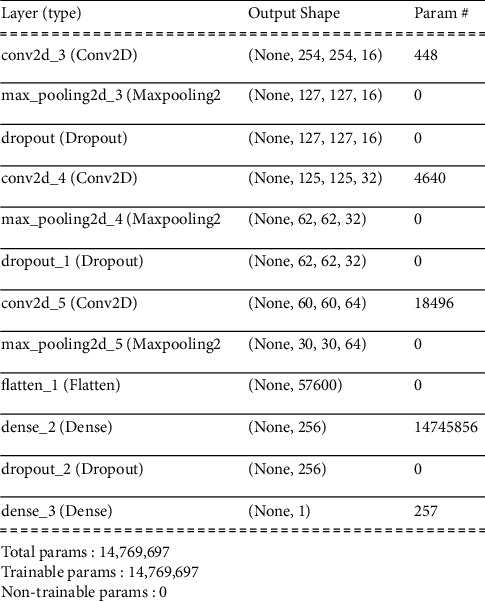
CNN model three architecture from summary ( ) function.

**Figure 5:**
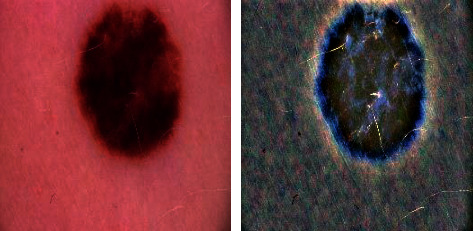
Before and after Gaussian function use.

In the preprocessing step, image quality was improved by removing noise using a Gaussian function.  Figure 4 shows before and after images demonstrating the effect of the Gaussian function.  Figure 5 shows the architecture of model 3.

#### 3.2.4. Fourth Model—VGGNet-16

VGG16 is a convolutional neural network model proposed by K. Simonyan and A. Zisserman [11] and was one of the most famous models submitted to ILSVRC-2014.

VGGNet-16 is a CNN architecture consisting of 16 layers composed of small convolutional filters. It also includes batch normalization, nonlinear activations with ReLU, and pooling layers after two or three convolutions. Then, 2 dense layers were applied, using different activation functions (rectified linear unit and sigmoid functions, in that order), giving us a model that is ready to compile. Adam was used as the optimizer with 0.0001 learning rate.

#### 3.2.5. Other Traditional Models

We also set up and applied other traditional (non-CNN) machine learning methods to our dataset, including support vector classification (SVC), K-nearest neighbor (KNN), Naïve Bayes, random forest (RF), and gradient boosting.

Using the integrated features of grid search provided in some of the methods, we were able to determine the best parameters to train our models more rapidly. Some of the methods did not have parameters to tune or did not have a well-functioning grid search implementation. The models that did have parameters have their values as shown in Table 4.

### 3.3. Results

**Figure 6:**
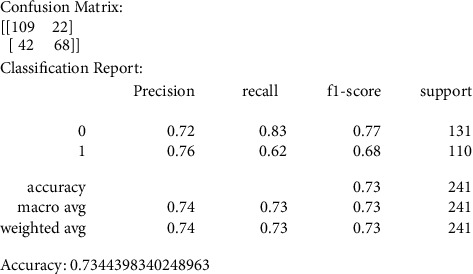
SVC confusion matrix, classification report, and accuracy.

**Figure 7:**
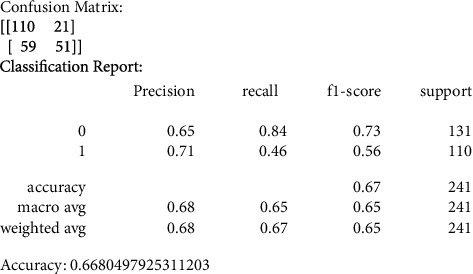
KNN confusion matrix, classification report, and accuracy.

**Figure 8:**
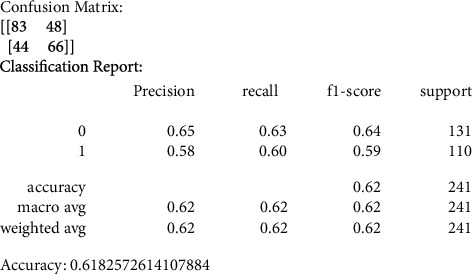
GNB confusion matrix, classification report, and accuracy.

**Figure 9:**
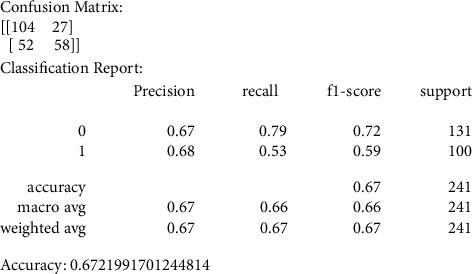
RF confusion matrix, classification report, and accuracy.

**Figure 10:**
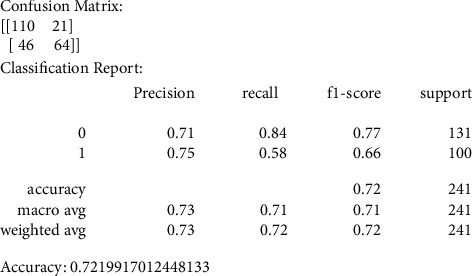
GB confusion matrix, classification report, and accuracy.

Our primary metric of performance is the level of accuracy achieved, by comparing to a control set of images that were not previously used in the training of the data models. Figures 6–10 present the confusion matrix, classification report, and accuracy of the traditional models we considered. Accuracies ranged from 61%–73% for the traditional models. While support vector classification yielded the highest accuracy of 73.44, the Naïve Bayes model yielded the lowest accuracy of 61.82%. Also, support vector classification has the highest precision and F1 score.

#### 3.3.1. Support Vector Classification (SVC)

The confusion matrix values resulting from the SVS model are represented by precision, recall, F1 score, and support metrics that are listed in Figure 6; we care about the accuracy average among other metrics.

#### 3.3.2. K-Nearest Neighbor (KNN)

We also produced the confusion matrix values for the KNN model; we noticed that the accuracy is less than what we obtained from the SVC model.

As shown in Figure 9, we noticed that random forest has performed a little better than KNN in terms of the accuracy average.

#### 3.3.3. Gaussian Naive Bayes (GNB)

Gaussian Naive Bayes supports continuous-valued features, and models conform to a Gaussian (normal) distribution. Therefore, an approach to creating a simple model is to assume that a Gaussian distribution describes the data with no covariance (independent dimensions) between dimensions.

#### 3.3.4. Random Forest (RF)

Random forest is a supervised learning algorithm that uses ensemble methods (bagging) to solve regression and classification problems. The algorithm operates by constructing a multitude of decision trees at training time and outputting the mean/mode of prediction of the individual trees.

#### 3.3.5. Gradient Boosting

Gradient boosting works by building simpler (weak) prediction models sequentially where each model tries to predict the error left over by the previous model. Because of this, the algorithm tends to overfit relatively quickly. But, what is a weak learning model? A model does slightly better than random predictions.

**Figure 11:**
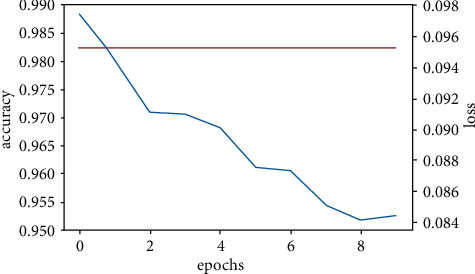
CNN model one accuracy and loss.

**Figure 12:**
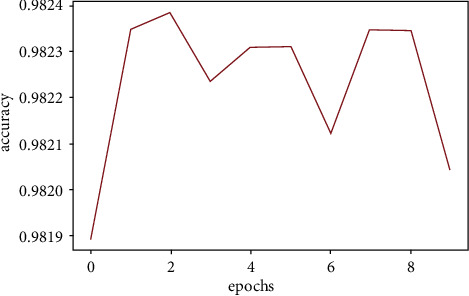
CNN model two accuracy fluctuation.

**Figure 13:**
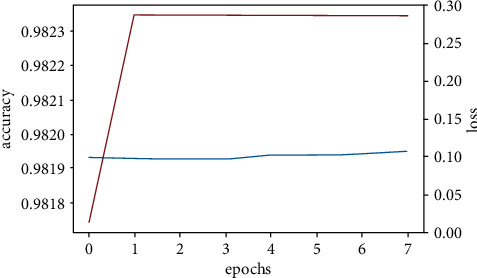
CNN model three accuracy fluctuation.

**Figure 14:**
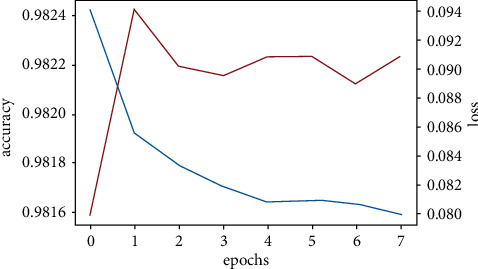
CNN model four accuracy and loss.

**Figure 15:**
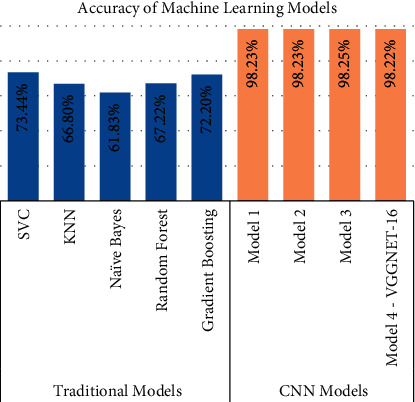
Comparison of different tested models and their overall accuracy.

We created three CNN models using different architectures (Experimental Section), calculated the accuracy, and compared them with the already available CNN model (VGGNet-16) (Figures 11–14). Overall accuracies of all four models as listed in Table 5 are estimated to be 98%, which are similar in all four models and significantly greater than the traditional classification models.  Figure 15 illustrates a comparison of overall accuracies of all the models we have considered. After optimization and fitting, CNN model accuracy of 98% was readily achieved and was relatively unaffected by manipulation of images in an attempt to improve model accuracy. Finally, we compared the execution time for machine learning algorithms used in this project as in Table 6.

#### 3.3.6. CNN Model One

The figure above represents the visualization of the accuracy and loss for the first CNN model which consists of 3 convolutional neural network layers of increasing kernel size, on a 3px by 3px.

#### 3.3.7. CNN Model Two

Figures 12 and Figure 13 represent the accuracy fluctuation for the second and third CNN models which focused on creating a normalized image.

#### 3.3.8. CNN Model Three

CNN model three accuracy fluctuation is shown in Figure 13.

#### 3.3.9. VGGNet

Figure 14 represents the accuracy and loss metrics for the fourth CNN model which used VGGNet and VGGNet-16.

#### 3.3.10. Overall Results

Table 5 compares the accuracy of the four CNN models created in this project.  Figure 15 used accuracy values to compare traditional models vs the four CNN models.

Finally, we compared the running time for all the machine learning algorithms we used in this project, and we listed them in Table 6.

## 4. Conclusion and Future Works

Based upon the results observed in the comparison of these models, it appears that using any of the implementations we created using a convolutional neural network model of machine learning has a significant improvement in accuracy.

The largest limitation of the works we have created is due primarily to the limited size of the dataset that was used. There are not many reliable sets of freely available data for skin imagery for use in research and development.

Possible applications of this work in the future could involve the inclusion of this model in automated diagnostic software, to enhance the diagnostic ability of both clinical dermatologists and oncologists.

This model could also be further extended by the inclusion of a larger dataset, possibly also making use of online learning, to create a model that would continually get better over time [12,13].

## Figures and Tables

**Table 1:** Comparison of machine learning algorithms from the related work section.

Article title and author	Method	Accuracy	Summarization
M-skin doctor: A mobile-enabled system for early melanoma skin cancer detection using a support vector machine Aleem et al. [3]	SVM	0.80	Aleem et al. published an article introducing a mobile-enabled cancer detection system for early melanoma skin cancer using a support vector machine (SVM). The proposed system can be identified as three main steps: preprocessing, segmentation, and feature extraction and classification. In the preprocessing step, image quality was improved by removing noise using the Gaussian function. In the segmentation step, the grab cut technique was used to split the image. In the feature extraction and classification step, meaningful features such as mean, standard deviation, and perimeter were extracted. They mainly choose histogram and ABCD features proposed by the ABCD rule. The SVM algorithm was applied as a classification technique. SVM algorithm provides good classification results in real-time smartphones. Even though the model has been only applied for skin melanoma, this application can be extended to other skin diseases (eczema and skin rashes). Its sensitivity and specificity rates are 80% and 75%. However, it would be worthwhile to evaluate the proposed system with a different algorithm such as CNN. The idea of using smartphone apps as cancer detection tools is explored, including the fact that at least 40 apps are already out that claim to do so. These tools can be harmful as they may not actually be using any sort of detection and may just be apps to track sizes of the lesion, etc., and do not have the typical protections in place that meet the requirements of medical information (HIPPA).

Melanoma detection byanalysis of clinical images using a convolutional neural network Esfahani et al. [4]	CNN	0.81	Clinical images (though not from a dermoscopy) were preprocessed to remove noise and illumination effects and fed into a convolutional neural network trained on many samples.

Expert-level diagnosis of nonpigmented skin cancer by combined convolutional neural networks. Tschandl et al. [5]	CNN	0.735	Tschandl et al. explored how CNN achieves professional-level accuracy in diagnosing pigmented skin cancer; however, most common types of skin cancers are nonpigmented and hard to diagnose. Thus, the author expected to compare the accuracy of a CNN-based classifier on the diagnosis of nonpigmented skin cancer with that of physicians with different levels of experience in this study. The proposed system can be identified as two main steps, such as neural network diagnoses and human rating. In the neural network diagnosis step, the first CNN-based classification model was trained on thousands of dermoscopic and close-up images of lesions removed at a primary skin cancer clinic for a combined evaluation of both imaging methods. The combined CNN (cCNN) was tested on a set of 2072 unknown cases and compared with the results from 95 human raters who were medical professionals with expertise in different areas of dermatology. CNN has achieved a higher percentage in nonpigmented skin cancer diagnosis than beginner and intermediate level medical personnel but not expert medical personnel. However, the presented model has a lower accuracy than other recent publications. This may be due to the small sample size with different classes, and using a large sample set could resolve this problem and improve the accuracy. Also, the proposed model did not evaluate with any other model.
The impact of patient clinical information on automated skin cancer detection Pacheco et al. [6]	ResNet-50	0.788	The article compares various methods of training a model to recognize cancer in images and considerations that must be made when doing so, particularly when it comes to unsupervised training. The most interesting point is that if control data images are taken on a different camera or dermoscopy, the model may end up learning to pick the images on the subtle differences in the image related to a given model of the device, not the cancer itself. This article goes into detail about one potential data source for images to be used for training, the International Skin Imaging Collaboration.
	ResNet-101	0.757	
	GoogleNet	0.779	
	MobileNet	0.762	
	VGGNet-13	0.746	
	VGGNet-19	0.750	

**Table 2:** Additional comparison for machine learning algorithms from the related work section.

Article title and author	Method	Accuracy	Summarization
Skins cancer identification system of HAMl0000 skin cancer dataset using convolutional neural network Nugroho, Ardan Adi, et al [7]	CNN	0.78	Nugroho et al. investigated to create a skin cancer identification system for decision making. The proposed system was based on the convolutional neural network (CNN) algorithm, and it has three stages such as convolutional layer, pooling layer, and fully connected layer. The convolution layer applies the output function as a feature map from the image. Rectified linear unit (ReLu) used as an activating function. Pooling layer was used to reduce the size of the representation and to reduce the speed. This layer mainly gives the ability to recognize an object. Fully connected layer is used to transform the data dimension and to connect the previous layer to the next layer. The results of this CNN model that uses input shape with the following parameters exhibit that the level of training accuracy is 80% and the testing accuracy is 78%; input shape size 90^*∗*^120-pixel, adam optimizer, learning rate 0.001, and number of epochs 50. Basal cell carcinoma disease was the most difficult to identify by the system, while actinic keratoses and intraepithelial carcinoma diseases are the most likely to be identified. However, the proposed model did not evaluate with any other model.

Recent advances in deep learning applied to skin cancer detection Pacheco, Andre G. C., and Renato A. Krohling. [8]	*n/a*	*n/a*	This article is a summary of how machine learning and image processing can help dermatologists more rapidly identify skin cancers, in particular melanomas (the deadliest form of skin cancer). Due to the pressures created by increases in healthcare cost, lack of qualified professionals, and lack of access to relevant medical tools, cases of melanoma being diagnosed at a late stage have been going up. The article explores solutions to this problem and makes three major arguments–images run through machine learning algorithms (particularly models made up of a composition of methods of learning) can be at least as effective at diagnosis of skin cancers as dermatologists (assuming a good image is given)–these algorithms need to be able to work with clinical image data (i.e., from standard cameras), rather than medical imaging devices, and that there is a significant lack of data for testing and training, particularly when it comes to data with relevant metadata (patient age, race, diseases, etc.) associated with an image. This article seeks to explore the basics of machine learning and how it can be applied to image processing, including examples of how it has already been applied in the field. As such, the main contribution to the field that this article has is as a compilation of works that have already been done at the intersection of machine learning and medical imagery. As such, the article has no new major contributions to add, as it is primarily derivative in nature, but is a good jumping-off point into the field of other works.

A convolutional neural network framework for accurate skin cancer detection Thurnhofer-Hemsi, K., Domínguez, E. [9]	DenseNet201	0.95	Another analysis was performed on the HAM10000 dataset using a DenseNet201 neural network and image augmentation, demonstrating that it may be an effective model to use for this purpose, due to its high classification accuracies and low rate of false negatives.

**Table 3:** Model one architecture.

Layer	Size	Output shape
Input shape	(256,256,3)	
Convolutional 2D + ReLu	16(3^*∗*^3) filter	(256,256,16)
Max pooling + ReLu	(2^*∗*^2) filter	(128,128,16)
Convolutional 2D + ReLu	32(3^*∗*^3) filter	(128,128,32)
Max pooling + ReLu	(2^*∗*^2) filter	(64,64,32)
Convolutional 2D + ReLu	64(3^*∗*^3) filter	(64,64,64)
Max pooling + ReLu	(2^*∗*^2) filter	(32,32,64)
Fully connected + ReLu	512 neurons	1
Fully connected + sigmoid	1	1

**Table 4:** List of parameters used for configuration of the traditional method of machine learning.

Model	Parameters
SVC	*C* = 3, Degree = 3, Gamma = auto, Kernel = rbf
KNN	Algorithm = auto, n_neighbors = 15, weights = distance
RF	Criterion = entropy, max_features = auto, n_estimator = 15
Gradient	Max_depth = 2 n_estimator = 50

**Table 5:** Our CNN models and their accuracy.

Model	Accuracy % with all benign
Model 1	98.23
Model 2	98.23
Model 3	98.25
Model 4—VGGNet-16	98.22

**Table 6:** Execution time comparison for machine learning algorithms.

Algorithm	Time taken for training (s)	Time taken for classification (s)
SVM	493.871434	0.154563
Naïve Bayes	0.453653	0.140434
Random forest with 2 trees	0.6941016	0.081072
Random forest with 5 trees	1.056321	0.126287
Random forest with 10 trees	1.520123	0.186565
Random forest with 20 trees	2.312458	0.349988
Random forest with 50 trees	4.965323	0.788574
CNN	6.358789	0.047896
KNN with *k* = 2	0	0.065487
KNN with *k* = 3	0	0.210213

## Data Availability

The data used to support the findings of this study are available from the corresponding author upon request.
